# Targeting Breast Cancer Stem Cells to Overcome Treatment Resistance

**DOI:** 10.3390/molecules23092193

**Published:** 2018-08-30

**Authors:** Sònia Palomeras, Santiago Ruiz-Martínez, Teresa Puig

**Affiliations:** New Therapeutic Targets Laboratory (TargetsLab) Oncology Unit, Department of Medical Sciences, University of Girona, Girona Institute for Biomedical Research, Emili Grahit 77, Girona 17003, Spain; sonia.palomeras@udg.edu

**Keywords:** BCSCs, resistance, targeted therapy, breast cancer, BCSCs markers

## Abstract

Despite advances in breast cancer diagnosis and treatment, many patients still fail therapy, resulting in disease progression, recurrence, and reduced overall survival. Historically, much focus has been put on the intrinsic subtyping based in the presence (or absence) of classical immunohistochemistry (IHC) markers such as estrogen receptor (ER), progesterone receptor (PR), and human epidermal growth factor receptor-related protein (HER2). However, it is widely understood that tumors are composed of heterogeneous populations of cells with a hierarchical organization driven by cancer stem cells (CSCs). In breast tumors, this small population of cells displaying stem cell properties is known as breast CSCs (BCSCs). This rare population exhibit a CD44^+^/CD24^−/low^ phenotype with high ALDH activity (ALDH^+^), and possesses higher tolerability to chemotherapy, hormone therapy, and radiotherapy and is able to reproduce the bulk of the tumor after reduction of cell populations sensitive to first-line therapy leading to disease relapse. In this review, we present special attention to BCSCs with future directions in the establishment of a therapy targeting this population. Drugs targeting the main BCSCs signaling pathways undergoing clinical trials are also summarized.

## 1. Introduction

Breast cancer (BC) is a prevalent human malignancy and a very common cause of cancer-related death among women worldwide. Currently, it is considered a multifactorial disease that involves an interaction between environmental, hormonal, and genetic influences, differences in lifestyle, or nutritional exposures. Therefore, patients with BC could have a wide range of clinical, pathological, and molecular characteristics [1]. 

BC has been classified in three categories according to clinical and histopathological characteristics and on the expression of estrogen receptor (ER), progesterone receptor (PR), and human epidermal growth factor receptor-related protein (HER2). The hormone-positive BC represents approximately 70% of BC patients and is characterized by the overexpression of ER and/or PR. Women with this kind of BC have a better prognosis than other cancer types [2]. The second subgroup is the HER2^+^ characterized by the overexpression and/or amplification of the human epithermal growth factor receptor 2 (HER2, also known as ErbB-2, ERBB2, or HER2/neu) [3,4]. It represents the 20% of BC patients and it is associated with a more aggressive phenotype and bad prognosis [3]. Meanwhile, the lack of expression of the aforementioned receptors give rise to the cancer known as Triple-Negative BC (TNBC) subgroup. TNBC represents 15–20% of the patients and it is described with an aggressive course and poor prognosis [5]. This histopathological classification is the most used but not the only one. Currently, five phenotypically distinct subgroups that correlate with clinical outcome have been identified including ER+/Luminal (luminal A and B [6]), basal-like, Erb-B2+, normal like, and the claudin-low based on the gene expression pattern has emerged [7,8]. This molecular classification is a complementary information for the conventional classification in tailoring treatment and predicting prognosis. These different BC subtypes and complexity within tumors remarks the heterogeneity of BC. Tumor is a complex structure with different clones of cancer cells and other cell types such as stromal, immune, or endothelial cells. Therefore, understanding this heterogeneity is necessary to be able to developed targeted therapies. 

For many years, it was considered that cancer was initiated due to an accumulation of genetic alterations in normal somatic cells which provided a selective advantage that drove the transformation of normal human cells into highly malignant derivatives [9]. However, during the last years the novel Cancer Stem Cell (CSC) theory changed that idea [10]. 

## 2. Cancer Stem Cells 

Stem cells are defined by three main properties: differentiation; self-renewal; and homeostatic control [11], and are essential for the proper functioning of the body acting as a source to replenish any tissue or organ cell throughout the life of a person with especial importance in the embryonic development. However, the same qualities in tumor development pose a huge challenge for oncologists and are a threat to the patients. Tumors are composed of heterogeneous population of cells among which, cancer stem cells (CSCs), a minority subpopulation of undifferentiated cells capable of giving rise to the differentiated cells that comprise the bulk of the tumor, are found. The CSC hypothesis claims that tumors, as well as normal tissues, are formed from a group of cells called cancer stem cells or “cancer-initiating cells” through asymmetric cell division, simultaneously maintaining the stem population and generating multi-lineage differentiation [12]. Nevertheless, the origin of these CSCs is still unclear and two models have been proposed. One model states that normal long-lived stem cells become malignant through the accumulation of genetic alterations while others believe that mutations may endow a lineage-committed cell with stem characteristics [13]. What seems to be increasingly clear is that CSCs are involved in tumor recurrence, metastatization, and drug resistance. The isolation of leukemic stem cells from acute myeloid leukemia was the first evidence supporting the hypothesis that tumors are organized in a hierarchical way with tumorigenic properties as seen using in vivo mouse models [14]. From that moment, CSCs have been isolated from a variety of cancer types, including brain [15], ovarian [16], lung [17], prostate [18], and breast cancer [19]. These tumorigenic cancer cells possess a specific profile of surface markers such as CD29, CD34^+^, CD38^−^, CD166, CD133^+/−^, Lin, stem cell antigen 1 (Sca-1), epithelial cell adhesion molecule or (EpCAM) associated with stemness and that may be used to isolate them by means of fluorescence activated cell sorting (FACS) or other immunoselection procedures [20,21,22]. The use of all the described surface markers must be done with caution since some can also be found in non-cancer stem cells [23,24]. Although we focus on the use of these markers as pharmacological targets, it must be said that they may be useful in prognosis, normally associated with more aggressiveness and invasiveness of the tumor. Subsequently to isolation, sphere-forming assays in serum-free medium with a combination of supplemental factors or serially transplantable in vivo tumors may be used to test stem characteristics of the selected cells and propagate CSCs. Other three-dimensional systems such as hydrogels, matrices composed by biological substances [25,26], or scaffolds, structures mostly made of biopolymeric material [27], have emerged as new strategies to increase the percentage of cells with stem characteristics. These facts have facilitated the study at molecular level of this rare population with the objective of identifying differences between normal stem cells as well as key dysfunctions implicated in stemness. To this end, three main CSC signaling pathways have been related to self-renewal and differentiation i.e., Notch, Wnt/β-catenin and Hedgehog (Hh) pathways. Other important signaling pathways in CSCs are the TNF-α/NF-κβ, transforming growth factor-β (TGF-β), receptor tyrosine kinase (RTK), and Janus kinase/signal transducer and activator of transcription (JAK-STAT) pathways [28,29]. Moreover, it has been demonstrated that the induction of epithelial-mesenchymal transition (EMT) resulted in cells with stem properties [30]. EMT is characterized by decreased E-cadherin expression in adherent junctions and upregulation of mesenchymal proteins (vimentin, N-cadherin, and fibronectin), endowing cancer cells with migratory, invasive, self-renewal, and drug resistance capabilities. Twist and Snail, two transcription factors involved in the E- to N-cadherin and in the repression of E-cadherin, respectively, are two of the most well-known EMT regulators. Other transcription factors involved in EMT include Slug, Zeb1, and Zeb2 [28]. The introduction of the CSC concept not only changes the idea of tumor initiation and maintenance but also sheds light on resistance mechanisms. Thus, this population should also be targeted in order to avoid resistance and recurrence acquired by current therapies, ideally leading to a complete response.

## 3. Breast Cancer Stem Cells (BCSCs)

**Figure 1:**
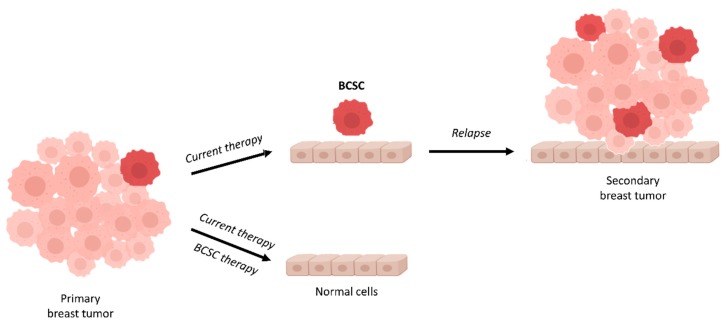
Schematic representation of tumor cell heterogeneity in an aggressive breast cancer. The primary tumor is formed of heterogeneous subpopulations of cells including breast cancer stem cells (BCSCs). Usually, a first-line therapy is chosen based on the histological subtype, stage of the tumor, presence of biomarkers, and other clinical data. If the first-line therapy does not target BCSCs, the mass of the tumor will be reduced but BCSCs will remain (mostly) unaffected. BCSCs present in the primary tumor can expand and give rise to a multi-drug resistant tumor (secondary breast tumor), leading to progression and metastasis of the tumor.

It has been identified a small population of CSC within BC termed Breast Cancer Stem Cells (BCSC). Although in recent years, BC treatment options have been increasing exponentially, the treatment resistance and different side effects increased the necessity to find new therapy treatments to overcome resistance in patients with this malignancy. BCSCs have been related to chemoresistance and BC relapse. It has been described that BCSCs survive treatment and are the cause of tumor relapse [31]. Therefore, a high proportion of BCSCs have been associated with poor outcome [32]. For this reason, many studies have focused on BCSCs analysis as well as on the identification of new drugs capable of eradicating this population (Figure 1). 

BCSCs were initially discovered in 2003 by Al-Hajj et al. [33]. Using cells from primary breast tumors and metastatic pleural effusions, they demonstrated that as few as a hundred cells expressing the adhesion molecule CD44, the epithelial surface antigen (ESA), and did not express CD24 (a ligand for P-selectin) or it was low-expressed (ESA^+^/CD44^+^/CD24^−/low^) were able to sustain growth when injected into mammary fat pads of non-obese diabetic severe combined immunodeficient (NOD/SCID) immunocompromised mice [33]. Conversely, even 100 times more of CD44^−^/CD24^+^ breast cancer cells injected to NOD/SCID mice failed to form tumors [33]. The BCSC population is not restricted to tumors or primary cells but also found in different established breast cancer cell lines, where isolated cells with a CD44^+^/CD24^−^/ESA^+^ phenotype (more correlated with basal than luminal breast cancer cell lines types) were able to reconstitute the parental cell line [34,35]. Established cell lines might be then a good cost-effective model to study BCSC characteristics and start developing drugs against this population. BCSCs can also be separated from other cell populations within the bulk of the tumor using the commercially available Aldefluor kit (Stem Cell Technologies, Inc.) based on aldehyde deshydrogenase (ALDH) activity. It is worth mentioning that even though ALDH^+^ cells have also been identified as tumor initiating cells, they partially overlap the isolated CD44^+^/CD24^−^ population, indicating that the phenotypically isolation of cells does not include all BCSCs [36]. Recent evidences suggest that BCSCs may form part of two interconvert dynamic mesenchymal-epithelial transition (MET)-EMT states that gives rise to more quiescent, mesenchymal-like BCSCs (CD44^+^/CD24^−^), localized at the tumor’s invasive front and more proliferative epithelial-like BCSCs (ALDH^+^), found more centrally within tumors [37]. This provides a logical explanation to the mismatch of phenotypically distinct population of cells with stem characteristics. The cell surface adhesion molecule CD44, is the hyaluronic acid (HA; also known as hyaluronan) receptor that mediates cell-cell and cell-extracellular matrix interactions. This transmembrane glycoprotein counts with various isoforms as a result of post-translational modifications such as N- and O-glycosylation and alternative splicing of the primary transcript, ranging from the standard isoform (CD44S) with none of the variable exons to all of the different isoforms containing exons v2–v10 (exon v1 is not expressed in humans) [36]. Eleonor Olsson and coworkers found a differential CD44 isoform pattern expression within several molecular subtype breast cancer cell lines, interestingly varying when the same cell line was cultured in adherent surface or in suspension as mammospheres, evidencing the importance of certain CD44v^+^ cell types in stemness [36]. Whereas, the small glycoprotein CD24, also known as heat stable antigen (HAS) is involved in the negative regulation of chemokine receptor CXCR4, which is involved in cellular adhesion, proliferation and metastasis. Although the CD44^+^/CD24^−/low^ markers have been widely used to identify breast cancer cells with stemness properties, there are controversies regarding their tumorigenicity. The controversy of these two cell markers led to seek new and more reliable stemness markers. Thus, CD133 or CD49f among others have been suggested. The transmembrane glycoprotein CD133 or Prominin 1 has been identified in many tumors including colon [38,39], liver [40], pancreas [41], or endometrium [42] among others. O’Brien et al. and Ricci et al. identified that CD133^+^ colon cancer cells had the ability to initiate tumor growth. In BC, although there are studies that identify the CD133 as a good BCSC marker [43,44], its prognostic role has not been well defined yet. The CD49f also known as integrin α6 (ITGA6) has been related to cell adhesion and signaling. CD49f has been associated to poor prognosis and reduced survival in BC [45]. 

**Figure 2:**
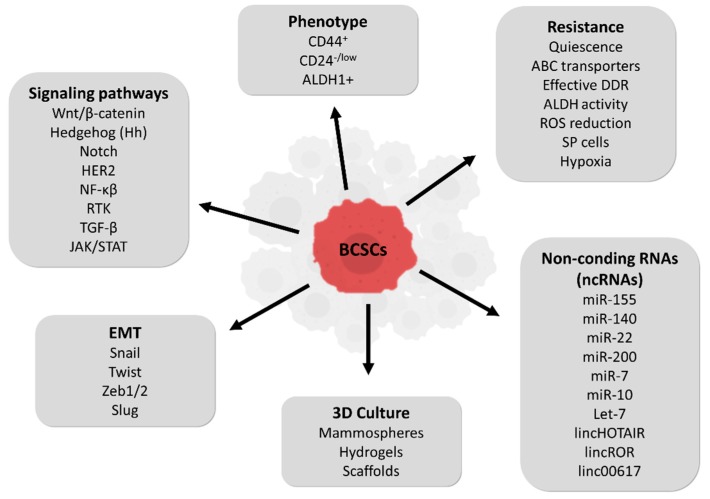
Summary of breast cancer stem cells (BCSCs) characteristics.

Additionally, BCSCs have also been seen to be TGF-β responsive and expressing the HER2 oncoprotein [34,46,47]. In contrast to differentiated cells that undergo anoikis, BCSCs have the ability to proliferate in suspension, being successfully propagated in vitro as nonadherent mammospheres through the mammosphere-forming efficiency (MSFE) assay [48,49]. Related to this, self-renewal ability is indirectly assessed by the ability of mammospheres to be serially passaged at a clonal density [48]. TGF-β1 exposure induces an EMT in non-tumorigenic, immortalized human mammary epithelial cell line (HMLEs), increasing by more than 40 times the mammosphere capacity of these cells. The same study proved the importance of the transcription factors Twist and Snail by ectopic expression of one of these two in HMLEs cells leading to significant increase in the number of mammospheres formed [30]. Recently, the JAK/STAT3 pathway has been identified to be important in BCSCs, the blockade of which leads to self-renewal inhibition and expression of lipid metabolic genes [50]. Analogously to other cancer type’s stem cells and in normal stem cells, in BCSCs the embryonic-related pathways Wnt/β-catenin, Hh and Notch, are dysregulated and play an important role in tumor resistance, recurrence and metastasis [51]. Wnt/β-catenin pathway has been involved in stemness and differentiation in various cancer cells. It has been described that BCSCs present a higher activity of Wnt pathway. Hh pathway has been associated with tumorigenesis and with maintenance of self-renewal in BCSCs [52,53]. Finally, Notch pathway is involved in cell fate regulation of the mammary gland [54] and has a critical role in BCSCs promoting chemoresistance and radioresistance [55] (Figure 2). These pathways in BC tumors are often altered by signals from tumor microenvironment or “niche” [56]. Analogously to stem cells and other CSCs, BCSCs interact via growth factors and cytokine networks with cells inside the tumor microenvironment, i.e., mesenchymal stem cells, tissue-associated fibroblasts, endothelial cells, adipocytes, and immune cells [57]. The intracellular communication within tumor microenvironment is through small vesicles called exosomes. These contain mRNAs, proteins, and non-coding RNAs (ncRNAs) that control gene expression of BCSCs. 

Recently, ncRNAs have been associated with BCSCs, mainly microRNAs (miRNAs) and long non-coding RNAs (lncRNAs). Different miRNAs such as miR-155 [58], miR-140, or miR-22 [59] have been specifically associated with the regulation of BCSCs properties, thus being useful as potential biomarkers and interesting targets to design a BCSC-directed therapy. Moreover, miR-200, miR-7, miR-10, and let-7 are different anticancer miRNAs related to drug resistance in stem cells [59,60]. Regarding lncRNAs, HOTAIR, ROR, and 00617 have been described to be involved in BCSCs by regulating EMT signaling pathways [61,62,63]. 

Therefore, the different BCSC properties identified are crucial for the development of new therapeutic targets for BC patients who progress to first-line treatment. 

## 4. BCSCs and Drug-Resistance

Standard therapies act on rapidly dividing cells and are in general effective in reducing the size of primary tumors, however complete tumor eradication is not always achieved due in part, by the presence of BCSCs. Although highly proliferative, BCSCs as well as their normal counterpart, remain most of the time in a quiescent state also known as dormancy (G0 phase of the cell cycle) that may be protecting them from chemotherapy and/or radiation damage [64]. Related to this, treatment with chemotherapy has been seen to increase the percentage of tumorigenic CD44^+^/CD24^−/low^ cells [31]. In addition, BCSCs express high levels of ATP-binding cassette (ABC) transporters, such as MDR1 (ABCB1) and the latest drug efflux pump discovered, BCRP (ABCG2) [65,66]. These cells known as “side population or SP” due to the ability of excluding dyes such as Hoechst 33342 or Rhodamine, are also capable of pumping out chemotherapeutic agents and represent 0.2–5.0% of the total cell population [67]. Interestingly, the SP phenotype is more commonly found in luminal subtype of BC compared to other subtypes [68]. Another stem cell indicator described is the ALDH enzyme which is important in stem cell differentiation via retinoic acid [69]. ALDH is related to resistance since it is able to metabolically inactivate chemotherapeutic agents such as cyclophosphamide [70]. It is worth mentioning that high ALDH activity might be due to the presence of different ALDH isoforms, however depending on the tumor type a specific isozyme will have a more relevant contribution [69]. For example, the ALDH isoform ALDH1 is a marker of normal and malignant mammary stem cells and ALDH1-positive tumors have proven to be more aggressive probably due to stem properties [47]. There is some evidence that ALDH1 depends on the signaling pathway PI3K/Akt of the HER2 receptor [71]. 

The relative resistance to radiation and cytotoxic agents of BCSCs may also be based in a more efficient mechanism of DNA damage response (DDR) which leads to a decrease in apoptosis compared to other mammary cell types [72] and the activation of DNA damage checkpoints genes. BCSCs expressing CD44^+^/CD24^-^ also have the capacity to reduce intracellular reactive oxygen species (ROS) levels induced in ionizing radiation, by overexpression of radical scavenger, by increasing the reduced glutathione synthesis though the Sx(-) antiporter system and by enhanced NADH and FADH2 products generated from metabolism alteration [50,73,74]. Additionally, BCSCs are predominantly located in hypoxic areas, which confers more radiotherapy and chemotherapy resistance. Hypoxia may increase the BCSC population through hypoxia-inducible factors (HIF-1α and HIF-2α) that promote cell dedifferentiation by upregulation of embryonic stem cell markers [75]. HIF expression and transcriptional activity induced after treatment with chemotherapy (paclitaxel or gemcitabine) in different human BC cell lines led to BCSC population enrichment through interleukin-6 (IL-6) and IL-8 signaling and MDR1 overexpression [76]. Antiangiogenic therapy (sunitinib and bevacizumab) also increased the proportion of BCSCs mediated through HIF-1α by the activation of the Wnt pathway via Akt/β-catenin signaling [77]. Other CSC intrinsic resistance mechanisms include the activation of PI3K/Akt signaling via phosphatase and tensin homolog (PTEN) driving cell cycle arrest [78], signaling activation of Wnt/β-catenin, Hh and Notch [79,80] and constitutive activation of NF-κB [81]. Recently, a study showed that cells resistant to trastuzumab expressed increased levels of Notch-1 which represses PTEN and leads to hyperactivation of ERK1/2 signaling [82] (for a summary see Figure 2).

## 5. Drugs Targeting (B)CSCs

The higher resistance of BCSCs to standard therapies in comparison with other cells of the tumor bulk highlights the need for new therapies targeting the stem population. All the aforementioned BCSCs properties, markers and mechanisms of resistance may be potential targets to designing a more efficient therapy to use alone or in combination with current used therapies, for the treatment of patients diagnosed with BC. 

The quiescent state of BCSCs might be altered by targeting the cyclin-dependent kinase inhibitors p57KIP2, p27KIP1, and p18INK4c as seen in hematopoietic (HCSs) [64,83] and chronic myeloid leukemia [84] stem cells. The authors proved the importance of these proteins in quiescence and self-renewal activity using knock-in mouse models. Fbxw7 (F-box protein), one of the four subunits of the SCF-type ubiquitin ligase complex, may be a potential target as it has been shown to be implicated in quiescence maintenance by reducing the levels of c-Myc, a transcriptional factor related to the transcriptional modulation of genes involved in cell cycle and proliferation functions. Fbxw7 ablation sensitize non-dividing leukemia-initiating cells to imatinib [85], therefore the stimulation of CSCs to re-enter the cell cycle may increase the effect of current therapies. Gasca et al. found that silencing Fbxw7 in TNBC (MDA-MB-468) endow this cell line with paclitaxel resistance and fbxw7 expression in cells resistant to the chemotherapeutic drug resensitized them [86]. Quiescence of HCSs might also be disrupted by overexpression of BCRA1 as seen in transgenic mice [87]. 

Sulfasalazine, an antirheumatic drug that inhibits the activity of the xCT protein, which forms the cysteine-glutamate transporter, reduced CD44v^+^ cells and GSH levels in tumor cells of patients with advanced gastric cancer [88], thus providing a rationale for therapy success improvement by increasing radiation sensitivity of CSCs, what in turn reduces the ability to compensate for ROS effects. The Ataxia Telangiectasia Mutated (ATM) gene constitutes the DNA damage surveillance/repair system. After radiation, CD44^+^/CD24^−^ BCSCs isolated from established breast cancer cell lines and primary culture of patient breast cancer cells presented high ATM signaling activity, and treatment with an ATM inhibitor resensitized these cells to radiation [89]. Thus, decreasing the radiation resistance of BCSCs by targeting the ATM signaling may prevent relapse after conventional first-line therapy. The stimulation of HIF-1α induced in hypoxia conditions that promotes BCSC development may be targeted by HIF-1α inhibitors such as ganetespib (a second-generation HSP90 inhibitor) [90] overcoming chemoresistance, both in vitro and in vivo [76]. In addition to HIF-1α inhibitors, other hypoxia-related strategies like the inhibition of the AlkB homolog 5 (ALKBH5) expression that depends on the hypoxia-inducible factors HIF-1α and HIF-2α, resulted in significant down-expression of NANOG, a pluripotency factor important in maintenance and specification of CSCs [91]. Other strategies involve the ALDH activity, which may also be targeted in combination with current therapy to increase better outcome of patients with BC. Alysha K. and Alison L. demonstrated that the inhibition of ALDH activity by means of all-*trans* retinoic acid (ATRA) or the specific ALDH inhibitor diethylaminobenzaldehyde (DEAB) increases the effect of chemotherapy (doxorubicin/paclitaxel) and radiotherapy on TNBC cells [92]. 

Salinomycin, an ionophore antibiotic isolated from *Streptomyces albus* used by veterinarians, has proven to selectively kill BCSCs in different histological types of breast cancer, by changing the expression of genes involved in metastasis-free survival, overall survival, tumorosphere formation ability, and EMT differentiation [55,93,94]. The combination of salinomycin targeting stem cells with current chemotherapeutic drugs i.e., doxorubicin or paclitaxel directed to cancer cells, common anti-HER2 targeted therapies (monoclonal antibody trastuzumab and the small molecule lapatinib), as well as a histone deacetylase inhibitor have synergistically inhibited tumor growth [93,95,96]. Enhanced cellular uptake and selectivity towards BCSCs of salinomycin has been achieved by using nanoparticles coated with HA, the primary CD44 binding molecule [94]. From fact, the function of CD44 expression as a hyaluronan receptor has been used to specifically direct drugs alone or encapsulated against the cancer stem population. A recent study showed that the used of hyaluronan-conjugated liposomes encapsulating the anticancer agent gemcitabine not only increased the inhibitory capacity of gemcitabine against BCSCs but also reduced the systemic toxicity of the drug alone on normal tissue, a fact to consider in the development of anticancer drugs [97]. Other strategies involving the CD44 are the inhibition of HA and its receptor by using small HA oligosaccharides that compete with endogenous HA polymer [98] or antibodies that block the HA-binding site of CD44 [99]. 

Dysregulated Wnt, Hh, and Notch signaling pathways have also been studied to establish pharmacological targets of BCSCs. Different dietary polyphenol compounds have been shown to directly or indirectly act on self-renewal and survival pathways of CSCs. Among them, sulforaphane from cruciferous vegetables [100,101], epigallocatechin-3-gallate, the most abundant catechin in green tea [102,103], resveratrol from red grapes, peanut, and blueberries [104,105], curcumin found in spices [106], and piperine from black and long peppers [106] have proven efficacy in targeting BCSCs. Interestingly, neither curcumin nor piperine affected differentiated cells while their effect to BCSCs was seen at relatively low concentrations, making both of them good candidates to be explored in combination with therapies targeting non-cancer stem cells. 

## 6. Drugs Targeting Wnt, Notch and Hh in Clinical Trials for Patients with BC

**Figure 3:**
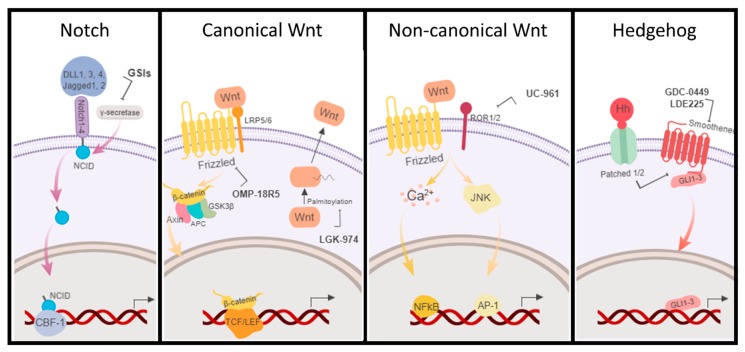
Schematic representation of the main BCSC signaling pathways, Notch, Wnt (canonical and non-canonical), and Hedgehog (Hh). Some of the current drugs in clinical trials directed to BCSC pathways are indicated. GSIs: γ-secretase inhibitors (MK-0752, RO4929097, and PF-03084014).

The CSC concept implies the development of new drugs targeting both CSCs and the bulk of the tumor or the combination of current therapies with CSC-targeted ones. Here we present the anti-BCSCs drugs developed targeting Wnt, Notch, and Hh pathways that have reached clinical trials for breast cancer patients (Figure 3). 

Notch counts with four transmembrane receptors (Notch1-4) that interact with five ligands (DLL1, 3, 4, Jagged1, 2). Due to this heterogeneity and the wide spectrum of possibilities, the most clinically evolved approach is the inhibition of Notch signaling using γ-secretase inhibitors (GSIs). Notch receptors are cleaved by γ-secretase, releasing the Notch intracellular domains (NCID) and subsequently activating Notch signaling. NCID is then translocated to the nucleus where it induces gene transcription by interacting with other co-factors. The experimental γ-secretase inhibitor MK-0752 (Table 1) from Merck in combination with docetaxel has reached phase I/II clinical trials for metastatic breast cancer. Undergoing serial patients’ biopsies showed a decrease in cell population with CD44^+^/CD24^−^ phenotype, ALDH^+^ activity and a reduction in MSFE, leading to the first evidence of the benefits of BCSC-targeted therapy thought the inhibition of Notch pathway in combination with systemic cytotoxic therapy [107]. Other GSIs for the treatment of breast cancer that have reached clinical trials are RO4929097 in combination with paclitaxel and carboplatin in patients with stage II/III TNBC (ClinicalTrials.gov Identifier: NCT01238133), PF-03084014, and LY450139 (semagacestat), the first GSI to enter phase III clinical trials for the treatment of Alzheimer’s Disease. CB-103 is a protein-protein interaction inhibitor targeting Notch signaling that is currently in phase I/II clinical trials for advanced or metastatic breast cancer (Table 1). 

BCSCs also show enhanced activation of Wnt pathway compared to the bulk of the tumor [108]. Wnt ligand may stimulate the canonical (Wnt/β-catenin dependent pathway) or two β-catenin-independent pathways, the Planar Cell Polarity (Wnt/JNK) pathway and the Wnt/Ca^2+^ pathway [109]. In the canonical pathway, the extra-cellular Wnt binds the heterodimer receptor form by the seven-pass transmembrane Frizzled (FZD) and the single-pass low-density lipoprotein LRP 5 or 6 which activates the complex made of Axin, Adenomatous polyposis coli (APC), and the glycogen synthase kinase 3β (GSK3β). Subsequently, the β-catenin is translocated to the nucleus where it binds to the TCF/LEF transcription factors and modulates the expression of Wnt-responsive genes. In the non-canonical pathway, the Wnt signal is mediated through FZD and/or ROR1/ROR2/RYK co-receptors, activating the JNK or Ca^2+^ signaling cascades [109]. Many studies have focused on different targets of the Wnt pathway such as PORCN, RSPO3, WNT2B, FZD5, FZD10, ROR1, tankyrase, and β-catenin, from which six drugs have been developed and reached clinical trials [110]. Among them, four molecules are or have been tested for the treatment of breast cancers, thus targeting BCSCs. Two monoclonal antibodies, vantictumab (OMP-18R5) and cirmtuzumab (UC-961), an anti-Frizzled and anti-ROR1, respectively, are studied in combination with paclitaxel for metastatic breast cancer (Table 1). There is now a clinical trial recruiting patients with breast cancer to find the recommended dose of LGK-974 (WNT974), an inhibitor of the endogenous Wnt palmitoleoylase PORCN, required for the palmitoylation of Wnt ligands, a necessary step in the processing of Wnt ligand secretion, alone and in combination with immunotherapy (an anti-PD-1). LGK-974 has proven efficacy in different cancer models both in vitro and in vivo [111]. Finally, two clinical trials have been started using Foxy-5, a formylated six amino acid peptide fragment that mimics the effects of Wnt5a, a non-canonical member of the Wnt family, which plays an important role in organ development, tissue orientation, cell polarity, and migration, thus, acting as an anti-metastatic cancer drug. 

The Hh pathway has also been explored in studies in vitro and in vivo. The Hh ligands (Sonic or Shh, Indian or Ihh, and Desert or Dhh) inhibit Hh pathway after binding to Patched 1 or 2, which in turns interacts with Smoothened that releases GLI1-3. Sims-Mourtada and co-workers co-treated breast cancer cells with Hh inhibitors and docetaxel and found a decrease in the CD44^+^/CD24^−^ BCSC population and mammosphere formation that was on the contrary increased when treating with docetaxel alone [112]. They relate the activation of Hh signaling with over expression of MDR1 and ABCG2 in BCSCs providing evidence for the inhibition of this pathway to avoid resistance to first-line therapy. Despite the existence of these pre-clinical promising results, only two Hh inhibitors have made their way to clinical trials for the treatment of breast cancer, both in combination with other drugs (Table 1). 

## 7. Conclusions

Increasing evidence indicates the existence of tumor initiating or cancer stem cells within tumors responsible in part of drug resistance and current treatment failure and recurrence. Significant advances have been made in the identification, isolation, and characterization of BCSCs and in consequence in the development of new compounds targeting this small cell population. All of the presented targets here might be of use for the development of a BCSC-directed therapy, however we believe a combination of the pharmacological targets would be desirable since BCSC resistant clones may appear because of the selection pressure derived from monotherapy. In like manner, the plasticity of BCSCs to shift between stem-like and non-stem-like states implies that the targeted therapy must not be restricted to this small population, but rather to a combination treatment also addressing more differentiated progenitors and the bulk tumor cell population.

## Figures and Tables

**Table 1:** Inhibitors of BCSCs signaling pathways in clinical trials.

Signaling Pathway	Drug	Phase	Status	Combined with:	Type of Breast Cancer	ClinicalTrials.Gov Identifier
**Notch**	MK-0752	Pilot	unknown	Tamoxifen or Letrozole	Early stage BC	NCT00756717
		I/II	completed	Docetaxel	Advanced or Metastatic	NCT00645333
		I	completed	NA	Metastatic or locally Advanced	NCT00106145
	RO4929097 (RG-4733)	I	completed	Letrozole	ER+, HER2−, PR+, stage II/IIIA	NCT01208441
		I	completed	Paclitaxel and Carboplatin	Stage II/III TNBC	NCT01238133
		I	completed	Vismodegib *	Metastatic TNBC	NCT01071564
		I	completed	Exemestane	Advanced or Metastatic	NCT01149356
		II	completed	NA	Advanced, Metastatic, or Recurrent TNBC	NCT01151449
		I	completed	Capecitabine	Refractory	NCT01158274
		I	completed	Cediranib Maleate	Advanced	NCT01131234
	PF-03084014 (Nirogacestat)	I	completed	Docetaxel	Advanced	NCT01876251
		II	withdrawn	NA	Chemoresistant TNBC	NCT02338531
		II	completed	NA	Advanced	NCT02299635
	LY3039478 (crenigacestat)	I	recruiting	Taladegib, Abemacinib, Cisplatin, Gemcitabine, Carboplatin, LY3023414	Advanced or metastatic	NCT02784795
	CB-103	I/II	recruiting	NA	Advanced or metastatic	NCT03422679
**Hedgehog**	GDC-0449 (vismodegib)	II	recruiting	Paclitaxel, Epirubicin, Cyclophosphamide	TN	NCT02694224
	LDE225 (sonidegib)	I	unknown	Docetaxel	Advanced	NCT02027376
		II	withdrawn	NA	Stage III ER-, HER2-	NCT01757327
		I	completed	BKM120	Metastatic	NCT01576666
**Wnt**	LGK-974 (WNT974)	I	recruiting	PDR001 (anti-PD-1)	TN	NCT01351103
	Foxy-5	I	completed	NA	Metastatic	NCT02020291
		I	recruiting	NA	Metastatic	NCT02655952
	OMP-18R5 (ventictumab)	I	completed	Paclitaxel	Metastatic	NCT01973309
	UC-961 (Cirmtuzumab)	I	not yet recruiting	Paclitaxel	HER2 negative metastatic	NCT02776917

* Inhibitor of Hedgehog pathway. TN: Triple negative; a breast cancer subtype HER2-, ER- (estrogen receptor) and PR- (progesterone receptor). NA: not applicable.
